# Long acting β2-agonist and corticosteroid restore airway glandular cell function altered by bacterial supernatant

**DOI:** 10.1186/1465-9921-11-6

**Published:** 2010-01-20

**Authors:** Jean-Marie Zahm, Franck Delavoie, Férial Toumi, Béatrice Nawrocki-Raby, Claire Kileztky, Jean Michel, Gérard Balossier, Malcolm Johnson, Christelle Coraux, Philippe Birembaut

**Affiliations:** 1 INSERM, U903, Reims, F-51092, France; 2 INSERM, U926, Reims, F-51097, France; 3 Univ Reims Champagne Ardenne, IFR53, Reims, F-51097, France; 4 CHU Reims, Laboratoire Pol Bouin, Reims, F-51092, France; 5 GlaxoSmithKlein Research and Development, Middlesex, UK

## Abstract

**Background:**

*Staphylococcus aureus *releases virulence factors (VF) that may impair the innate protective functions of airway cells. The aim of this study was to determine whether a long-acting β_2 _adrenergic receptor agonist (salmeterol hydroxynaphthoate, Sal) combined with a corticosteroid (fluticasone propionate, FP) was able to regulate ion content and cytokine expression by airway glandular cells after exposure to *S. aureus *supernatant.

**Methods:**

A human airway glandular cell line was incubated with *S. aureus *supernatant for 1 h and then treated with the combination Sal/FP for 4 h. The expression of actin and CFTR proteins was analyzed by immunofluorescence. Videomicroscopy was used to evaluate chloride secretion and X-ray microanalysis to measure the intracellular ion and water content. The pro-inflammatory cytokine expression was assessed by RT-PCR and ELISA.

**Results:**

When the cells were incubated with *S. aureus *supernatant and then with Sal/FP, the cellular localisation of CFTR was apical compared to the cytoplasmic localisation in cells incubated with *S. aureus *supernatant alone. The incubation of airway epithelial cells with *S. aureus *supernatant reduced by 66% the chloride efflux that was fully restored by Sal/FP treatment. We also observed that Sal/FP treatment induced the restoration of ion (Cl and S) and water content within the intracellular secretory granules of airway glandular cells and reduced the bacterial supernatant-dependent increase of pro-inflammatory cytokines IL8 and TNFα.

**Conclusions:**

Our results demonstrate that treatment with the combination of a corticosteroid and a long-acting β_2 _adrenergic receptor agonist after bacterial infection restores the airway glandular cell function. Abnormal mucus induced by defective ion transport during pulmonary infection could benefit from treatment with a combination of β_2 _adrenergic receptor agonist and glucocorticoid.

## Background

The epithelial lining of the airways provides an efficient barrier against microorganisms through interdependent functions including mucociliary clearance, homeostasis of ion and water transport, biochemical responses and acts as a cellular barrier function by means of intercellular junctions. These functions are fundamental to the maintenance of the defence and the integrity of the airway epithelium which may be disturbed after any infectious insult in diseases such as chronic obstructive pulmonary disease (COPD) or cystic fibrosis (CF). *Staphylococcus aureus *(*S. aureus*) is one of the most common gram-positive bacteria involved in airway infections, either primary or subsequent to viral diseases [1]. *S. aureus *is also a major cause of hospital acquired lower respiratory tract infections and is often implicated in early infectious airway disease in CF patients [2]. *S. aureus *expresses several potential virulence factors (VF) that may induce airway epithelium injury and impair the epithelial wound/repair process [3]. Remodeling that occurs following injury may considerably disturb the innate protective function of the respiratory epithelium. Abnormal expression and distribution of CFTR protein is not only caused by mutations of the CF gene but is also observed in non-CF inflamed and/or remodeled airway tissues [4] and may thereby induce alteration of the airway mucus mainly produced by the airway glandular cells [5,6]. Abnormal mucus production is the hallmark of chronic inflammatory airway diseases such as asthma, chronic bronchitis, and CF [7,8]. Sputum has altered macromolecular composition and biophysical properties which vary with disease, but unifying features are failure of mucociliary transport resulting in airway obstruction [9]. Protection of the airway epithelium or restoration of its function requires factors that prevent or reverse cellular damage caused by bacterial VF. There is already evidence of enhanced respiratory cytoprotection against bacterial infection when airway epithelial cells are pre-incubated with a long-acting beta-2 adrenergic receptor (β_2_AR) agonist [10]. Furthermore, the increased CFTR expression associated with β_2_AR stimulation may have other beneficial effects on ion and water transport, protein expression and differentiation [11]. We have also shown that pre-treatment with the combination of a long-acting β_2_AR (salmeterol hydroxynaphthoate, Sal) and a corticosteroid (fluticasone propionate, FP) induces a downregulation of *S. aureus*-induced airway epithelial inflammation, particularly by modulating the expression of cytokines such as IL-6, IL-8 or TNFα [12].

Although previous studies have shown a preventive role of combined β_2_AR agonist/corticosteroid (Sal/FP) on COPD exacerbations [13] and bacterial VF-induced alterations in human airway epithelial cells, the role of this combination used as a treatment to correct the deleterious effect of bacterial VF is currently unknown. In addition, whether bacterial infection of airway epithelial cells may induce alterations in ion transport and loss of epithelial electrolyte homeostasis has not been extensively investigated. Therefore, the aim of this study was to determine whether Sal/FP combination is able to restore intracellular ion and water content and inflammatory cytokine expression previously altered by *S aureus *supernatant. The experiments were performed on an airway glandular cell line since these cells are the main source of airway mucus and associated secretion products (ions, mucins, cytokines,) [6]. In addition these cells are characterized by numerous intracellular secretory granules which can be analyzed in terms of ion concentration. Since *S. aureus *VF have been demonstrated to be able to disrupt actin cables [14] and that this disruption may lead to CFTR delocalisation [15], we also investigated the effect of Sal/FP treatment on actin and CFTR cellular localisation. The use of Sal/FP combination is based upon experiments by which tissues incubated with low concentrations of Sal/FP would support a synergistic action between the two compounds and that the higher concentrations showed no added benefit with respect to mucosal damage compared to either agent alone at the same concentration [16].

Our results demonstrate that *S. aureus *VF produced during airway infection induce alterations of ion and water content in airway secretory granules, which may be at the onset of decreased mucociliary clearance frequently observed during pulmonary infection exacerbations [17]. Treatment with a corticosteroid combined with a β_2_AR agonist is able to correct these anomalies and may be helpful for restoring normal cytoprotective properties of the airway epithelium.

## Methods

### Preparation of bacterial supernatant

*S. aureus *strain 8325-4, a wild-type laboratory strain (fibronectin-binding protein (FnBP) A^+ ^and FnBPB^+^, NTC 8325 cured of prophages), was a generous gift from T.J. Foster (Department of Microbiology, Trinity College, Dublin, Ireland). Bacterial supernatant was prepared by growing bacteria in trypticase soy broth (TSB, AES Laboratoire, Bruz, France) for 16-18 h at 37°C under agitation (120 rpm). Supernatant of 5 × 10^8 ^cfu/ml was obtained by centrifugation at 960 *g *for 10 min at 4°C, then filtration through a 0.2 μm filter (Pall Gelman Science, Ann Arbor, Michigan). The supernatant containing *S. aureus *soluble VF was diluted to 2%, 10% or 20% in Dulbecco's modified Eagle's medium (DMEM)/F-12 (Sigma Aldrich, St Louis, MO). TSB was used as control at 2, 10 or 20% in DMEM/F-12.

### Preparation of salmeterol hydroxynaphthoate and fluticasone propionate

Salmeterol hydroxynaphthoate (Sal), provided by GlaxoSmithKline (Uxbridge, UK), was dissolved in a minimum amount of glacial acetic acid (30 μl), then diluted to a concentration of 2 × 10^-4 ^M in phosphate-buffered saline (PBS; Gibco, Invitrogen, Paisley, UK) and kept at -20°C. The solution was buffered to a pH of 7.4. The stock solution was used at a final concentration of 2 × 10^-7 ^M in DMEM/F-12 previously defined as optimal for inducing airway epithelial cytoprotection [18].

A stock solution of fluticasone propionate (FP) provided by GlaxoSmithKline was prepared (1 × 10^-5 ^M) in 1 mM ethanol (Merck Eurolab, Darmstadt, Germany). FP was diluted with DMEM/F-12 medium to a final concentration of 1 × 10^-8 ^M, a concentration previously found to have anti-inflammatory effects in bronchial epithelial cells [19].

### Cell culture and experimental procedure

The transformed human tracheal glandular cell line MM-39 [20] was grown in DMEM/F-12 supplemented with 1% Ultroser G serum substitute (Biosepra, Villeneuve-la-Garenne, France), glucose (10 g/l), sodium pyruvate (0.33 g/l), penicillin (100 IU/ml), streptomycin (100 μg/ml) and amphotericin B (2 μg/ml) on porous membranes (12-well Transwell Clear, Costar, France) coated with type I collagen (50 μg/ml) prepared as previously described [21] and was cultured at 37°C under a 5% CO_2 _atmosphere. In a first set of experiments to determine the effect of *S. aureus *supernatant on cell death, cells were incubated with 2%, 10% or 20% of *S. aureus *supernatant or with medium alone (DMEM/F-12 supplemented with 2%, 10% or 20% of TSB) for 5 hours. In the subsequent experiments, cells were incubated with either control medium alone (DMEM/F-12 supplemented with 2% of TSB), or in presence of *S. aureus *supernatant (2%) for 1 h, then treated with Sal/FP (2 × 10^-7 ^M and 1 × 10^-8 ^M, respectively) or vehicles (glacial acetic acid and ethanol) for 4 h.

### Assessment of cell viability

A fluorescence staining method using propidium iodide and syto9 (Molecular Probes, Eugene, OR) was used to study the cell death/viability of airway epithelial cells incubated with *S. aureus *supernatant. Propidium iodide only penetrates into cells with damaged membranes, staining the cells in red, whereas syto9 penetrates into all cells, staining them in green. Briefly, at cell culture confluence, medium was removed from the culture plates, and cells were washed three times with sterile PBS and incubated with 2%, 10% or 20% of *S. aureus *supernatant or TSB, propidium iodide (1 μl/ml) and syto9 (1 μl/ml). Culture dishes were placed on the stage of an inverted microscope (Axiovert 200 M; Zeiss, Le Pecq, France) equipped with an environmental chamber (37°C, 5% CO_2_, 100% relative humidity) and with a charge-coupled device video camera (Coolsnap Fx; Roper Scientific, Tucson, AZ). Using Metamorph (Universal Imaging, Downingtown, PA) software, we recorded time-lapse fluorescent images every hour for 5 h. Variations of the fluorescence intensity of propidium iodide were related to the variation of the number of dead cells. To assess the cell viability, airway glandular cells were seeded on a 96 well microplate. At confluence, they were incubated for 5 h with 2%, 10% or 20% of *S. aureus *supernatant or TSB in culture medium and then for 1 hour with 1 mg/ml methylthiazolyldiphenyl-tetrazolium bromide (MTT, Sigma Aldrich, St Louis, MO). The dye was extracted with propanol-2 and the OD at 560 nm was read in a Xenius spectrophotometer (Safas, Monaco).

### Western blot analysis

For membrane extract, 2% *S. aureus *supernatant-treated or 2% TSB-treated cells were disrupted mechanically in cold Tris buffer (50 mM Tris-HCL pH 7.5, 1 mM EDTA with complete protease inhibitor mixture (Roche Applied Science) for 15 min on ice and precipitated at 4°C overnight with 4% (v/v) trichloroacetic acid. After centrifugation (2500 *g *for 10 min at 4°C), the pellet was dissolved in 100 μl RIPA buffer. Six μg of protein extracts were separated by electrophoresis on 7.5% SDS-polyacrylamide gels and electroblotted to PVDF membranes using 100 V for 1 h at 4°C. Membranes were incubated for 1 h in a blocking buffer containing 5% non-fat dry milk in PBS with 0.1% Tween 20, then overnight with mouse anti-CFTR antibody (clone 24-1, 1:1000, R&D Systems, Lille, France) or with rabbit anti-actin antibody (A2066, 1:1000, Sigma-Aldrich, St Louis MO, USA) and finally with horseradish peroxidase (HRP)-conjugated anti-mouse immunoglobulin antibody (1:1,000; DakoCytomation, Glostrup, Denmark) or horseradish peroxidase (HRP)-conjugated anti-rabbit immunoglobulin antibody (1:1000, DakoCytomation). Blots were revealed by using an ECL+ kit (GE Healthcare, Little Chalfont, UK) and analyzed by densitometry with a Fuji Las-1000 (Raytest, Courbevoie, France).

### Actin and CFTR co-localisation by immunocytochemistry

To detect actin by immunofluorescence, we used an affinity isolated antigen specific antibody obtained from rabbit anti-actin antiserum by immuno-specific purification (A2066, 1:25, Sigma-Aldrich, St Louis MO, USA) [22]. CFTR was detected using the MAB25031 antibody (clone 24-1, diluted 1:100, R&D Systems, Lille, France) which is recommended by the European Working Group on CFTR expression [23]. MM-39 cells were seeded onto glass slides coated with type I collagen (50 μg/ml) and fixed at confluence with cold methanol for 10 min at -20°C. After sequential incubation with the anti-actin antibody, Alexa Fluor 594-conjugated goat anti-rabbit antibody (1:200, Molecular Probes, Eugene, OR), anti-CFTR antibody and Alexa Fluor 488-conjugated goat anti-mouse antibody (1:200, Molecular Probes), cells were incubated for 10 min with DAPI (4',6'-diamino-2-phenylindole, 200 ng/ml, Sigma Aldrich) for nuclear staining, then mounted with Aquapolymount antifading solution (Polysciences, Warrington, Pennsylvania) onto glass slides. Slides were observed under an AxioImager fluorescence microscope (Zeiss, Le Pecq, France) equipped with an apotome device (Zeiss). Images were recorded with a CCD video camera (Coolsnap, Roper Scientific, Tucson, AZ) at 40 successive z levels (0.25 μm between each z level) at ×63 magnification. The Metamorph software (Universal Imaging, Sunnyvale, CA) was used to quantify regions of overlap of actin and CFTR fluorescence. Both source images were thresholded and the areas of overlap were determined by calculating the number of pixels of actin staining overlaping with CFTR staining. Data were expressed in percentage of pixel overlap.

### Measurement of chloride efflux

The chloride efflux in airway epithelial cells was evaluated by videomicroscopy using the halide-quenched dye 6-methoxy-N-(3-sulfopropyl) quinolinium probe (SPQ, Molecular Probes) in a chloride buffer solution (130 mM NaCl, 2.4 mM K_2_HPO_4_, 10 mM D-glucose, 1 mM CaSO_4_, 1 mM MgSO_4_, and 10 mM Hepes) made hypotonic by adding an equivalent volume of water, as previously described [24]. Thereafter, the hypotonic chloride buffer was replaced by an isotonic chloride buffer for 15 minutes and then by a nitrate buffer in which the NaCl was replaced by 130 mM of NaNO_3_. The culture dish was placed on the heated stage of an inverted microscope (TE 300; Nikon, Champigny sur Marne, France). After 30 seconds, amiloride (10 μM), and 1.5 minutes later, forskolin (25 μM), were added to the nitrate buffer. Throughout the experimental process, fluorescence images λ_ex _at 365 nm and λ_em _at 395 nm) were recorded every 15 seconds using a Micromax CCD camera and the Metafluor software (Roper Scientific, Evry, France). Chloride efflux was calculated by measuring the variations in SPQ fluorescence (ΔF/Δt) over a 1.5 min incubation period after the addition of forskolin, and expressed as arbitrary units. In some experiments, the cells were incubated for 1 h in serum-free culture medium containing 5 μM CFTR_inh-172 _(Sigma Aldrich), which is a thiazolidinone CFTR inhibitor [25].

### Ion and water content analysis

Ion and water content was determined using electron probe X-ray microanalysis and a quantitative dark field intensity technique with a scanning transmission electron microscope (STEM CM30, Philips) for measuring the *in situ *ion and water content in the cytoplasm and in the secretory granules of entire cryofixed cells [26]. In practice, the cryosection of cells irradiated by an electron beam emits an X-ray signal. The emission spectrum corresponds to the counting of X-rays emitted according to their energy. The intensity ratio specific peak/background allows the measurement of the concentrations of all the elements detected in the spectrum. To obtain the exact value of the mass concentrations (mmol/kg dry weight) of the elements of interest (Na, Mg, S, Cl and K), we measured under the same experimental conditions, the specific peak/background ratio of the elements compared with standard samples of known mass concentrations. The mass concentrations in mmol/kg of dry matter (Cd) can be converted into mmol/l of water (C_h_) by using the equation C_h _= ((100 - L)/L) × Cd where L is the percentage of water determined by quantitative dark field imaging. The water mass content was deduced from the complement to 100% of dry mass content measured on the dark field images. We developed an original method for intracellular water content quantification with high spatial resolution (< 30 nm) based on dark field imaging. A hydrated cryosection contains a dry mass percentage (M) and its water complement (L) with L + M = 100%. During biological sample freeze-drying inside the microscope column, water (under amorphous ice sate) is sublimed and then the relative dark field intensity becomes directly proportional to the percentage of sample dry mass. By image processing, we obtained a parametric image in which the grey levels were proportional to the mass water content (L). The intracellular water content (L) was calculated by comparison with relative dark field intensities of standard samples with known water content. According to the different experimental conditions, 36 to 65 secretory granules from 14 to 21 cells were analyzed. Prior to the quantitative X-ray microanalysis, we showed that the K/Na ratio in the nucleus and in the cytoplasm was higher than 5, which is a characteristic of living confluent cells [27].

### Cytokine secretion measurement

Culture medium was collected and cytokine protein levels were determined using sandwich enzyme-linked immunoabsorbent assays (ELISA) for IL-8, IL-6 and high-sensitivity TNFα detection (R&D Systems, Minneapolis, MN) following the manufacturer's instructions. Results are expressed as pg/ml.

### RNA extraction and Reverse Transcriptase-Polymerase Chain Reaction analysis

RNA extraction of cells was performed with the High Pure RNA isolation kit (Roche Diagnostics GmBH, Mannheim, Germany) following the manufacturer's instructions. Reverse transcriptase (RT)-polymerase chain reaction (PCR) was performed with 10 ng of total RNA using the GeneAmp Thermostable RNA PCR Kit (Perkin Elmer, Foster City, CA) and three pairs of oligonucleotides (Eurogentec, Seraing, Belgium). Forward and reverse primers for human IL-8, TNF-α, and 28 S were designed as follows: IL-8 primers, forward 5'-GCCAAGGAGTGCTAAAGAACTTAG-3', reverse 5'-GAATTCTCAGCCCTCTTCAAAAAC-3'; TNF-α primers, forward 5'-CAGCCTCTTCTCCTTCCTGA-3', reverse 5'-TGAGGTACAGGCCCTCTGAT-3' and 28 S primers, forward 5'-GTTCACCCACTAATAGGGAACGTGA-3', reverse 5'-GGATTCTGACTTAGAGGCGTTCAGT-3'. For the IL-8 PCR, an initial denaturation at 95°C for 2 min was followed by 25 amplification cycles (denaturation at 94°C for 15 sec, annealing at 60°C for 20 sec, and elongation at 72°C for 10 sec) and a final 2-min elongation at 72°C. For the TNF-α PCR, the conditions were as follows: initial denaturation (94°C, 2 min), 29 amplification cycles (denaturation 94°C, 30 sec, annealing 59°C, 30 sec, and elongation 72°C, 30 sec) and final elongation (72°C, 7 min). For the 28 S PCR, the conditions were as follows: initial denaturation (95°C, 2 min), 13 amplification cycles (denaturation 94°C, 15 sec, annealing 66°C, 20 sec, and elongation 72°C, 10 sec), final elongation (72°C, 2 min). The expected sizes of the transcripts of IL-8, TNF-α and 28 S were 222 bp, 302 bp and 212 bp, respectively. RT-PCR products were separated by acrylamide gel electrophoresis, stained with SYBR gold (Molecular Probes) and visualized by fluorimetric scanning (Fuji, LAS-1000, Raytest, France). The IL-8 and TNF-α mRNA values were normalized to 28 S mRNA values. Results represent the mean ± SD of eight independent experiments performed in duplicate.

### Data analysis

Values were reported as mean ± SEM. Non parametric Man and Whitney test and one-way Kruskall-Wallis test were used for comparisons between groups and differences were considered to be statistically significant with P values less than 0.05.

## Results

### Effect of *S. aureus *supernatant on cell viability

**Figure 1:**
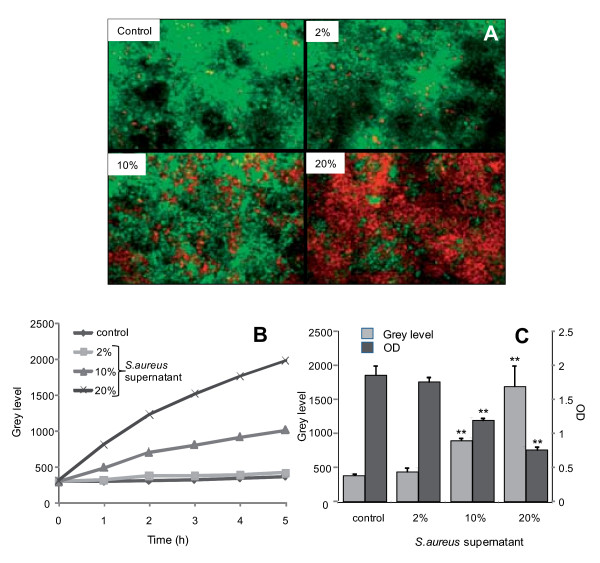
**Effect of *S. aureus *supernatant on cell death**. Cell death induced by *S. aureus *supernatant. (A) Propidium iodide fluorescent probe (red staining) was used to visualize the dead cells and syto 9 fluorescent probe (green staining) was used to visualize all the cells. The number of dead cells was increased in presence of 10% or 20% of *S. aureus *supernatant. (B) Time-dependent increase in fluorescence intensity of propidium iodide in presence of the different concentrations of *S. aureus *supernatant. (C) Fluorescence intensity of the propidium iodide probe after 5 h of incubation with the different concentrations of *S. aureus *supernatant. The increase in fluorescence was significant when the cells were incubated with 10% or 20% of *S. aureus *supernatant (*, p < 0.05; data represent the mean ± SEM of 3 different experiments). In parallel, the MTT technique showed the number of living cells. The decrease of OD was significant when the cells were incubated with 10% or 20% of *S. aureus *supernatant (**, p < 0.01; data represent the mean ± SEM of 8 different wells).

To assess the effect of *S. aureus *supernatant on cell viability, airway glandular cells were incubated with increasing concentrations of bacterial supernatant. Cell death was evaluated by using the propidium iodide fluorescent probe and cell viability by using the MTT assay. Figure 1A displays fluorescent images recorded after 5 hours of incubation with *S. aureus *supernatant. In control condition or in presence of 2% *S. aureus *supernatant, a limited number of cells showed a red nucleus staining characteristic of dead cells, whereas the number of dead cells dramatically increased in presence of 10% or 20% of *S. aureus *supernatant. A typical time-dependent increase in red fluorescent staining is displayed in figure 1B, showing a similar curve pattern for the control experiment and the experiment in presence of 2% *S. aureus *supernatant. The comparison of the grey levels of the red fluorescence after 5 h of incubation with *S. aureus *supernatants is shown in figure 1C. A significant increase in fluorescence, reflecting the increase in cell death, was observed when the cells were incubated with 10% or 20% of *S. aureus *supernatant (p < 0.01). In parallel, using the MTT technique, we quantified by OD measurement the number of living cells and observed that this number significantly (p < 0.01) decreased in presence of 10 or 20% of *S. aureus *supernatants (figure 1C). From these data we can therefore conclude that the incubation for 5 h with 2% *S. aureus *supernatant did not significantly altered the cellular viability.

### Effect of *S. aureus *supernatant on CFTR expression

**Figure 2:**
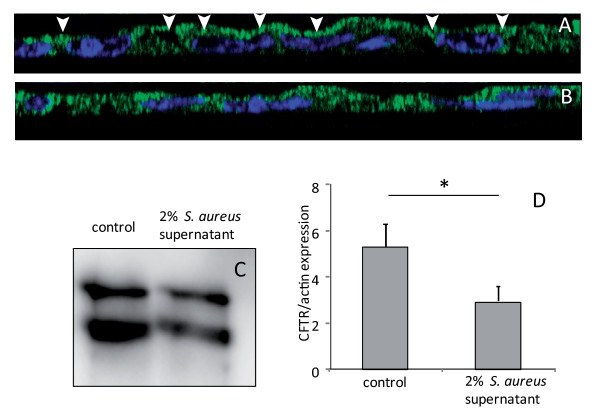
**Effect of *S. aureus *supernatant on CFTR localisation and expression**. (A, B) Immunolocalisation of CFTR (green staining) and Dapi nuclei staining (blue) in lateral view of successive z level images. In control cells, we noticed an apical staining of CFTR (arrow heads in A). In 2%*S. aureus *supernatant-treated cells (B), the CFTR staining was more diffuse in the cytoplasm. (C) Western blotting analysis of airway glandular cell membrane proteins showed the presence of CFTR in control cells and in fewer amount in cells incubated with 2%*S. aureus *supernatant. (D) Quantitative measurement showed a significant (*, p < 0.05) decrease in CFTR expression in cell membranes when cells were incubated with 2% *S. aureus *supernatant. Data represent the mean ± SEM of 5 different experiments.

Immunofluorescence and Western blotting were used to test the effect of *S. aureus *supernatant on CFTR expression at the cell membrane level. As shown in figure 2, we observed that the incubation of airway glandular cells with 2% *S. aureus *supernatant reduced the expression level of CFTR at the cell membrane, indicating a delocalisation of this protein from the apical membrane. CFTR localisation was assessed by using immunofluorescence imaging at different z levels. Figure 2A and 2B shows the cellular distribution of CFTR in a lateral image obtained from different z levels. In the control cells (figure 2A) we observed a high staining at the apical pole of cells. In the cells treated with 2% *S. aureus *supernatant, we observed the loss of the CFTR staining at the apical pole and a more diffuse cytoplasmic staining. We performed complementary western blotting analysis on cell membrane extracts (figure 2C) and measured a significant (p < 0.05) decrease in CFTR expression when the cells were incubated with 2% *S. aureus *supernatant (figure 2D).

### Actin and CFTR co-localisation is restored by Sal/FP treatment

**Figure 3:**
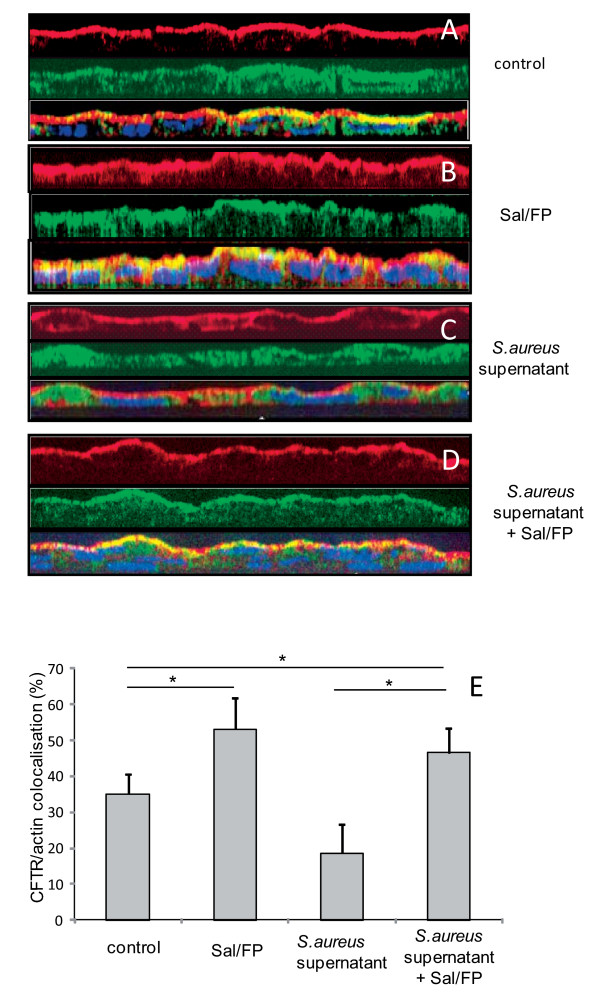
**Co-localisation by immunofluorescence of CFTR and actin**. (A) The pattern of CFTR (green staining) and actin (red staining) stainings was essentially apical in control cells as well as in cells treated with Sal/FP (B). (C) The incubation of cells with *S. aureus *supernatant induced alteration of the localisation of CFTR that appeared to be cytoplasmic, in parallel with a disorganization of the actin network. (D) Treatment of *S. aureus *supernatant pre-incubated cells with Sal/FP restored CFTR and actin apical stainings. (E) Quantification of the co-localisation of CFTR and actin showed that 2% *S. aureus *supernatant decreased the co-localisation index compared to the index in control cells, but the difference was not significant; the treatment with Sal/FP alone or after *S. aureus *supernatant incubation significantly enhanced the co-localisation of the 2 proteins compared with control or with *S. aureus *supernatant-treated cells (*, p < 0.05). Data represent the mean ± SEM of 3 different experiments.

Since it has been previously demonstrated that CFTR may directly bind actin and that this interaction may affect the functional properties of this channel protein [28], we aimed at analyzing the effect of *S. aureus *supernatant on actin and CFTR relationship. For that purpose, we examined the co-localisation of these proteins by immunofluorescence. The pattern of staining of CFTR (green staining) and actin (red staining) was essentially apical in control cells (figure 3A). The incubation of cells with Sal/FP enhanced the apical localisation of CFTR (figure 3B). In contrast, incubation of cells with 2% *S. aureus *supernatant induced an alteration in the localisation of CFTR which appeared to be more cytoplasmic (figure 3C) as previously shown in figure 2. Treatment of cells with Sal/FP restored CFTR and actin apical localisation (figure 3D). Quantification of the co-localisation of CFTR and actin (figure 3E) showed that 2% *S. aureus *supernatant decreased by 47% the co-localisation index compared with control cells, but the difference was not statistically significant. Interestingly, treatment with Sal/FP alone or after *S. aureus *supernatant incubation significantly enhanced the co-localisation of the 2 proteins compared with control cells (p < 0.05) or with *S. aureus *supernatant-treated cells (p < 0.05).

### *S. aureus *supernatant altered chloride efflux, ion and water content

**Figure 4:**
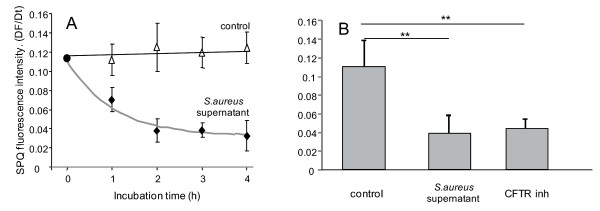
**Effect of *S. aureus *supernatant on cAMP-mediated chloride efflux**. (A) Time-dependent effect of *S. aureus *supernatant incubation on cAMP-mediated chloride efflux. We observed a significant (p < 0.01) time-dependent decrease in chloride efflux when cells were incubated with 2% *S. aureus *supernatant. This decrease became significant as soon as after 1 hour of incubation with 2% VF. (B) CFTR_inh172 _significantly decreased the chloride efflux compared to control (**, p < 0.01) and this decrease was similar to the decrease induced by *S. aureus *supernatant. Data represent the mean ± SEM of 3 different experiments.

We next analyzed the time-dependent effect of 2% *S. aureus *supernatant incubation on cAMP-mediated chloride efflux, and on cytoplasm and secretory granule ion and water content in airway epithelial cells. As shown in figure 4A, a significant (p < 0.01) time-dependent decrease in chloride efflux was observed after 4 hours of incubation with 2% *S. aureus *supernatant. This decrease became significant after 1 hour (36%) and reached 70% after 4 h incubation. To test whether the effect of *S. aureus *supernatant on chloride secretion was specific to CFTR function alteration, we compared the effect of *S. aureus *supernatant with the effect of a CFTR inhibitor. We observed that incubation of airway glandular cells with the CFTR inhibitor significantly reduced (p < 0.01) the chloride secretion and that this decrease was similar to the decrease observed after 4 h of incubation with 2% *S. aureus *supernatant (figure 4B).

**Figure 5:**
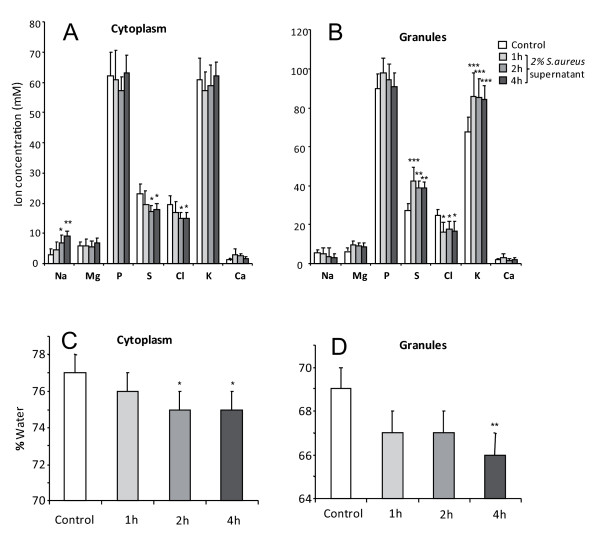
**Time-dependent effect of *S. aureus *supernatant on the ion and water content**. (A) We observed a significant (*, p < 0.05) time-dependent increase in sodium concentration and decrease in sulfur and chloride concentrations in the cell cytoplasm. (B) In the secretory granules, *S. aureus *supernatant incubation induced a significant increase in sulfur and potassium concentrations (*, p < 0.05; **, p < 0.01) and in parallel a significant (*, p < 0.05) decrease in chloride concentration. (C) The water content was significantly decreased in a time-dependent way by *S. aureus *supernatant in the cytoplasm and (D) in the secretory granules (*, p < 0.05). Data represent the mean ± SEM from 36 to 65 cytoplasmic areas or secretory granules from 14 to 21 cells.

Figure 5 shows the time-dependent effect of *S. aureus *supernatant on the ion concentration and water content measured either in the cell cytoplasm or in the secretory granules. After 2 hours of incubation with *S. aureus *supernatant, we observed a significant (p < 0.05) increase in sodium concentration and a decrease in sulfur and chloride concentrations in the cytoplasm (figure 5A). In the secretory granules, 1 hour of incubation with *S. aureus *supernatant induced a significant increase (p < 0.05) in sulphur and potassium concentrations and in parallel a significant (p < 0.05) decrease in chloride concentration (figure 5B). The water content was significantly decreased in the cytoplasm (figure 5C, p < 0.05) and in the secretory granules (figure 5D, p < 0.01) after 2 and 4 hours of incubation with *S. aureus *supernatant, respectively.

### Sal/FP restores chloride efflux, and ion and water content

**Figure 6:**
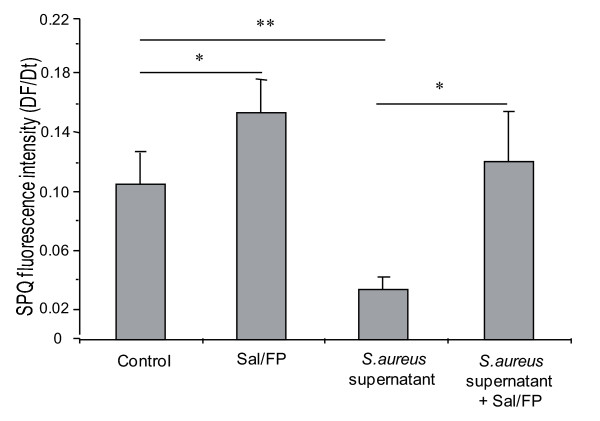
**Effect of Sal/FP treatment on the chloride efflux**. A significant decrease (*, p < 0.05) in chloride efflux after 1 h incubation with 2% *S. aureus *supernatant was observed. The treatment of airway epithelial cells with Sal/FP significantly (*, p < 0.05) restored the chloride efflux previously decreased by *S. aureus *supernatant. Data represent the mean ± SEM of 3 different experiments.

**Figure 7:**
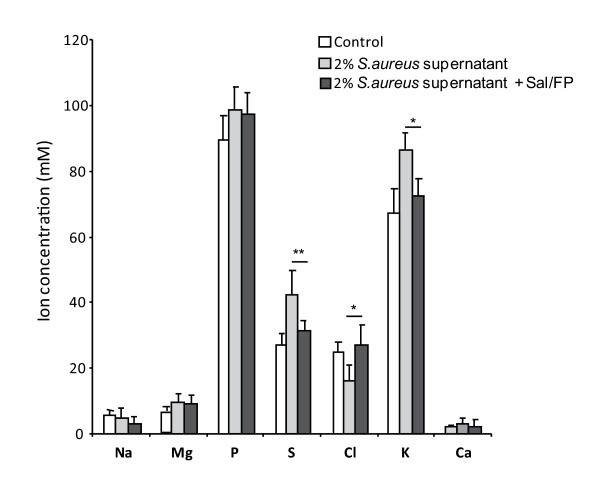
**Effect of Sal/FP treatment on the ion content**. We observed that Sal/FP treatment significantly increased the chloride content (*, p < 0.05) and decreased the sulfur and potassium content (**, p < 0.01 and *, p < 0.05, respectively) in the intra-cytoplasmic secretory granules, compared to *S. aureus *supernatant-treated cells. Data represent the mean ± SEM from 36 to 65 cytoplasmic areas or secretory granules from 14 to 21 cells.

We analyzed the effect of Sal/FP combination on the chloride efflux and ion and water content in airway epithelial cells. The cells were incubated for 1 h with 2% *S. aureus *supernatant and then with Sal/FP for 4 h. As shown in figure 6, we observed a significant decrease (p < 0.01) in chloride efflux after 1 h incubation with 2% *S. aureus *supernatant compared to control cells. Incubation of airway epithelial cells with Sal/FP restored the chloride efflux previously decreased by *S. aureus *supernatant. Interestingly, incubation of cells with Sal/FP alone significantly (p < 0.05) enhanced the chloride efflux. We observed that Sal/FP treatment did not significantly modify the cytoplasmic ion and water content (data not shown), but significantly increased the chloride content (26 ± 4 mM *versus *16 ± 5 mM p < 0.05) and decreased the sulfur (31 ± 2 mM *versus *42 ± 6 mM, p < 0.01) and potassium (72 ± 3 mM *versus *86 ± 8 mM, p < 0.05) content in the secretory granules, compared with the *S. aureus *supernatant-treated cells (figure 7).

### Sal/FP treatment downregulates *S. aureus *supernatant-induced airway epithelial cytokine release

**Figure 8:**
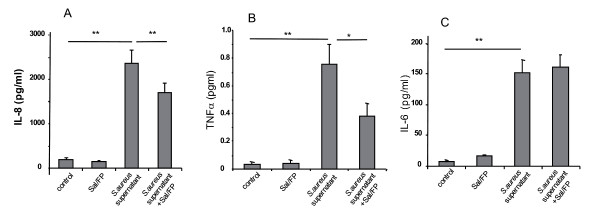
**Analysis of IL-8, IL-6 and TNFα protein secretion by ELISA**. A 1 h incubation with 2% *S. aureus *supernatant significantly (**, p < 0.01) increased IL-8, IL-6 and TNFα secretion when compared to control condition (A, B and C, respectively). When incubation with 2% *S. aureus *supernatant for 1 h was followed by incubation with Sal/FP for 4 h, *S. aureus *supernatant-induced IL-8 (A) and TNFα (B) secretion was significantly (**, p < 0.01, *, p < 0.05, respectively) decreased while Sal/FP had no effect on *S. aureus *supernatant-induced IL-6 secretion (C). Data represent the mean ± SEM of 8 (A and B) or 6 (C) independent experiments performed in triplicate.

We investigated whether following the incubation of airway epithelial cells with *S. aureus *supernatant, treatment with Sal/FP was able to modulate cytokine release. Incubation of epithelial cells with *S. aureus *supernatant for 1 h induced a 12-fold, 21-fold and 21-fold increase (p < 0.01) in the release of IL-8, TNFα and IL-6, respectively, compared with control cells (figure 8A, B and 8C). Interestingly, following 1 h incubation of epithelial cells with *S. aureus *supernatant, a 4 h Sal/FP treatment significantly (p < 0.01) reduced the *S. aureus *supernatant-induced IL-8 release (28%, figure 8A). Sal/FP treatment also decreased (p < 0.05) *S. aureus *supernatant-induced TNFα secretion (50%, figure 8B) whereas it had no effect on *S. aureus *supernatant-induced IL-6 release (figure 8C).

### Sal/FP treatment downregulates S. aureus supernatant-induced airway epithelial cytokine mRNA levels

**Figure 9:**
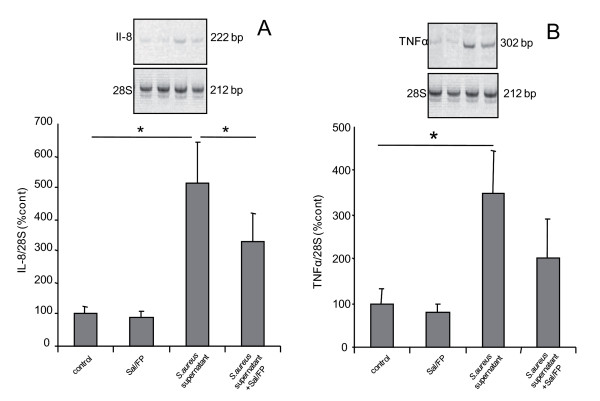
**Semi quantitative RT-PCR analysis of IL-8 and TNFα mRNA levels**. The incubation with *S. aureus *supernatant for 1 h increased significantly (*, p < 0.05) IL-8 and TNFα mRNA levels compared to control condition (A and B, respectively). When the incubation of *S. aureus *supernatant for 1 h was followed by treatment with Sal/FP for 4 h, *S. aureus *supernatant-induced IL-8 (A) mRNA level was significantly (*, p < 0.05) reduced, but no significant effect was observed for TNFα. Results are normalized to the 28S level and are expressed as mean ± SEM of 8 independent experiments performed in duplicate.

We next determined by semi-quantitative RT-PCR the effects of Sal/FP on *S. aureus *supernatant-induced IL-8 and TNFα mRNA levels. As shown in figure 9, incubation of epithelial cells with *S. aureus *supernatant for 1 h induced a 5-fold and a 3.5-fold increase (p < 0.05) in IL-8 (figure 9A) and TNFα mRNA (figure 9B) levels, respectively, compared with control cells. Following 1 h incubation of epithelial cells with *S. aureus *supernatant, Sal/FP treatment for 4 h significantly (p < 0.05) reduced *S. aureus *supernatant-induced IL-8 mRNA level (36%, Figure 9A), but had no significant effect on TNFα(figure 9B).

## Discussion

In the present study, we show that the treatment of airway epithelial cells with a combination of a corticosteroid and a long-acting β_2_AR agonist, after incubation with *S. aureus *supernatant, restores the function of airway glandular epithelial cells previously altered by bacterial VF. We pre-incubated airway glandular cells with crude extracts from *S. aureus*, which contain many types of VF including toxins and proteases. The main purpose of the present work was to evaluate the effect of drugs able to restore the airway epithelium functions rather than to pinpoint which bacterial factors are responsible for the alterations of these functions. We have chosen to test the effect of the combination of Sal and FP since it has been previously demonstrated that this combination induced a marked increase in the nuclear glucocorticoid receptor expression in airway epithelial cells and a significant synergistic decrease of IL-8, IL-6 and TNF-α, at both transcriptional and translational levels [12].

It has been suggested by Nadel and Borson [29] that ion transport in airways can be severely altered during infection and inflammation. Indeed, Swiatecka-Urban *et al *[30] reported that a cell-free filtrate of *Pseudomonas aeruginosa *reduced CFTR-mediated transepithelial chloride secretion by inhibiting the endocytic recycling of CFTR. Our results are in accordance with recent studies which reported that recombinant sphingomyelinase C (membrane-damaging virulence factor originally called β-hemolysin) from *S. aureus *strongly inhibited CFTR-dependent chloride current and that the cytoskeleton was remodelled through the acid sphingomyelinase/ceramide pathway [31,32]. Moreover, it has been previously demonstrated that actin cytoskeleton organization was required for cAMP-dependent activation of CFTR [33,34]. It is likely that the decreased activity of CFTR observed in presence of *S. aureus *supernatant could be related to the disruption of the actin cytoskeleton, leading to delocalisation and consequently inhibition of CFTR as demonstrated here by immunofluorescence. Glucocorticoids have been shown to increase the stability of actin filaments, increase actin polymerization, activate cytoskeleton-associated kinases and stabilize actin filaments against disruption by injury [35]. We hypothesize that incubation of *S. aureus *supernatant-treated cells with FP might prevent actin cytoskeleton degradation, leading to the recovery of functional CFTR chloride channels. In addition to the effect of FP on CFTR function, Taouil *et al *[11] previously demonstrated that the β2-AR agonist Sal was able to increase CFTR expression in human airway epithelial cells. It is also known that actin can interact directly or indirectly with epithelial ion channels through scaffolding proteins (NHERFs) or actin-binding proteins. Ganeshan *et al *[36] demonstrated that CFTR surface expression and chloride current were decreased by inhibitors of actin polymerisation. Together, these data indicate that modulation of the actin cytoskeleton may be a mechanism for regulating the CFTR function. Our findings also support the hypothesis that infection alters airway epithelial ion transport and that combination treatment with glucocorticoids and long-acting β2-AR agonists may be helpful in restoring normal epithelial ion transport function.

At the cytoplasmic level, we observed that *S. aureus *supernatant induced an increase in sodium concentration, which reflected an inability to regulate sodium absorption, likely related to a reduced CFTR function at the apical membrane. The reduced CFTR function is likely linked to CFTR delocalisation as assessed by immunocytochemistry. As a biological significance, one can compare this 3-fold increase in sodium concentration to the 5-fold increase that we observed when comparing sodium content in secretory granules from non-CF and CF cells [37]. This increase was accompanied by a decrease in cytoplasmic chloride concentration, despite the defect of cAMP CFTR-mediated chloride secretion assessed by fluorescence microscopy. Since there was an increase in cytoplasmic calcium concentration, we can therefore hypothesize that calcium activated chloride channels (CaCC) could be involved. Increased cytosolic calcium in host cells can be induced by bacterial toxins [38]. We can therefore speculate that *S. aureus *supernatant may be responsible for the time-dependent increase in calcium concentration, followed by the upregulation of CaCC and, as a consequence, an activation of calcium-dependent chloride conductance. The increase of sulfur content in the secretory granules after incubation with *S. aureus *supernatant could be associated with increasing mucin synthesis observed in previous studies [39]. All these data suggest a general mechanism by which epithelial glandular cells respond to the presence of bacteria.

In a previous study [12], we have demonstrated that *S. aureus *supernatant induced an upregulation of pro-inflammatory molecules such as IL-8, IL-6 and TNFα at mRNA and protein levels. We also reported that the pre-incubation of airway cells with the combination of Sal and FP had a beneficial effect in downregulating inflammatory cytokines following *S. aureus *infection. Pro-inflammatory mediator levels are markedly increased in induced sputum of patients with COPD [40]. The concentration of these mediators are further increased during acute exacerbations [41] and a correlation has been reported between pro-inflammatory mediator concentrations and the bacterial colony count in sputum [42]. In our present study, we confirm that *S. aureus *supernatant, even at low concentration, induces a marked increase in IL-6, IL-8 and TNFα release by airway glandular cells. Elevated TNFα levels are associated with increased IL-8 levels, and TNFα is a major inducer of IL-8 expression in lung epithelial cells [43]. In addition, TNFα which is increased by *S. aureus *supernatant might also act on the regulation of CFTR function. Indeed, it has been reported that this cytokine was able to modulate ion secretion in different kind of epithelia. Although it increases ion secretion in human intestinal epithelium [44], TNFα attenuates β agonist-evoked increase in chloride secretion across canine tracheal epithelium [45]. Using epithelial cells expressing wild-type CFTR or mutant CFTR lacking its PDZ-interacting domain, Dudez *et al *[46] have demonstrated that TNFα increased the amount of wild-type CFTR but not mutant CFTR in detergent-resistant membrane microdomains in which CFTR interacts with TNFα receptor-1 signaling cascade to regulate cytokine expression. Alteration of the formation of this complex may provide an explanation to some dysfunction of epithelial cell lacking a functional CFTR as observed in our study or in CF. Some studies have shown that β2-AR agonists, when given on their own, increased IL-8 release, but a strong synergistic inhibitory interaction between β2-agonists and steroids on IL-8 production has been described [47]. Steroids increase β2-receptor synthesis and β2-agonists prime glucocorticoid receptors for steroid-dependent activation and enhance nuclear translocation [48]. Interestingly, post-treatment with the combination of Sal/FP significantly reduced the IL-8 and TNFα level compared with levels in *S. aureus *supernatant-treated cells.

## Conclusion

The present data demonstrate that bacterial infection may lead to severe disruption of ion transport across the airway epithelium and to associated secretory granule dehydration, which may lead to defective mucociliary clearance in the airways, as previously shown by Puchelle *et al *who reported a relationship between the degree of infection and the rheological and transport properties of airway mucus in CF [8]. Together, our data indicate that abnormal mucus and upregulation of inflammatory cytokines associated with defective ion transport could benefit from treatment with drugs that could restore a normal function of secretory airway cells.

## Competing interests

JMZ has received research grants from GSK (UK) to support work carried out in the laboratory. MJ is employed by GlaxoSmithKline who markets Seretide, a combination of a long-acting beta-agonist (salmeterol) and a corticosteroid (fluticasone propionate). FD, FT, BNR, CK, JM, GB, CC, PB do not have a financial relationship with a commercial entity that has an interest in the subject of this manuscript.

## Authors' contributions

JMZ developed image analysis techniques, drafted the manuscript and participated in its design and coordination. FD carried out X-ray microanalysis and water content measurement. FT performed ELISA and RT-PCR for cytokine analysis. CK participated in cell culture and immunocytochemistry. BNR performed western blot analysis. JM, GB, MJ, CC and PB conceived the study, participated in its design and coordination and helped to draft the manuscript. All authors read and approved the final manuscript.
